# Influence of nitrogen water interaction on leaf functional traits of dominant species in warm temperate forest

**DOI:** 10.48130/forres-0024-0006

**Published:** 2024-03-15

**Authors:** Wen Li, Mingyang Liu, Mengke Li, Ruomin Sun, Tenglong Zhou, Yaqi He, Jianing Mao, Chang Liu, Lei Ma, Shenglei Fu

**Affiliations:** 1 College of Geography and Environmental Science, Henan University, Jinming Avenue No. 1, Kaifeng 475004, PR China; 2 Dabieshan National Observation and Research Field Station of Forest Ecosystem at Henan, Kaifeng 475004, PR China; 3 Key Laboratory of Geospatial Technology for Middle and Lower Yellow River Regions (Henan University), Ministry of Education, Kaifeng 475004, PR China; 4 Xinyang Academy of Ecological Research, Xinyang 464000, PR China

**Keywords:** Canopy nitrogen addition, Canopy water addition, Nitrogen water interaction, Leaf functional traits, Community weighted mean

## Abstract

Plant functional traits are indicative of plant responses to environmental changes, influencing ecosystem functions. Leaves, as a key focus in studying plant functional traits, present an area where the impact of nitrogen deposition and altered rainfall patterns on functional diversity remains ambiguous. To elucidate plant response mechanisms to environmental factors, we employed a canopy-based platform to add nitrogen, water, and their combination. We assessed the functional traits and community-weighted mean of the leaves of three dominant trees and three dominant shrubs. The results showed that nitrogen addition to the canopy significantly increased the leaf dry matter content of the* Celtis sinensis* Pers, but markedly decreased the specific leaf area of the *Liquidambar formosana* Hance. The nitrogen-water interaction did not notably affect the specific leaf area and equivalent water thickness of leaves. Canopy addition of nitrogen, water, and their combined interaction substantially lowered leaf nitrogen content and markedly increased leaf C/N. The structural equation model demonstrated a significant negative correlation between leaf dry matter content, equivalent water thickness, and leaf nitrogen content, as well as between equivalent water thickness and leaf phosphorus content. Our results provide evidence for the adaptation of plants to the environment and different strategies for resource and energy utilization.

## Introduction

In recent years, there has been heightened awareness regarding the surge in atmospheric nitrogen (N) deposition^[1]^. This excessive accumulation can adversely impact ecosystems. Concurrently, a notable uptick in precipitation has been documented globally, a phenomenon largely ascribed to climate change^[2,3]^. These concurrent shifts in N deposition and precipitation regimes are significantly altering the dynamics of temperate forest ecosystems. These alterations can precipitate changes in leaf functional traits, which are pivotal in modulating ecosystem processes such as productivity, nutrient cycling, and hydrological balance^[4,5]^. Comprehending these intricate interactions is vital for forecasting forest ecosystem responses to imminent environmental shifts.

Leaves as the primary interface for plant-environment exchanges , characterized by rapid growth and a high turnover rate rapid growth and high turnover rate^[6]^. Even minor variations in leaves growth can greatly affect ecosystem carbon dynamics and nutrient cycling, thereby contributing to feedback loops that affect global climate patterns^[7]^. Leaf growth in forests is particularly sensitive to fluctuations in N and water availability, given that N and water are the key factors for plant growth and development^[8]^. Consequently, an enhanced understanding of leaf responses to changes in N and water availability is essential for predicting their impact on the carbon dynamics within forest ecosystems and the feedback on global change. Leaves are also primary producers for plant organic material production, and leaf functional traits are acutely responsive to environmental shifts^[9]^. Understanding how leaf functional traits vary under different environmental conditions is crucial for exploring plant adaptation to environmental changes^[10]^. In the current context of climate change, studying the plasticity and variability of leaf functional traits can infer plant adaptation strategies and prospective evolutionary trajectories.

Previous research has demonstrated that, under variable regional conditions, plant species exhibit distinct leaf functional trait responses to N deposition due to differing environmental adaptation mechanisms. Traits such as specific leaf area (SLA), leaf dry matter content (LDMC), equivalent water thickness (EWT), leaf N content (LNC), and leaf phosphorus content (LPC) respond uniquely to N deposition across species^[11−13]^. In N-deficient desert ecosystems, modest N fertilization can significantly boost LNC, thus facilitating plant growth^[14]^. In subtropical evergreen broad-leaved forests, N deposition minimally influences LPC but markedly enhances leaf carbon and N content^[15]^. Additionally, N deposition levels also affect the accumulation and absorption efficiency of N in plants^[5]^. For pine leaves, under high N deposition levels, SLA significantly decreases, while LNC significantly increases under moderate N deposition levels^[10]^. Increased precipitation can profoundly influence forest leaf functional traits, affecting how trees interact with their environment^[16]^. One immediate impact of heightened rainfall is the potential for larger leaf sizes, as increased water availability supports cellular expansion and growth^[17]^. Conversely, some species may evolve leaves with quicker water-shedding attributes, such as modified leaf angles or pronounced drip tips, in response to frequent rainfall^[18]^. The increased water availability can also result in thinner leaves with lower leaf mass per area, which might be more efficient in light capture and gas exchange^[19]^. However, existing studies on the effects of N and water addition on leaf functional traits in forest ecosystems have not yet converged on a consensus. The interaction between increased N deposition and precipitation on forest leaf functional traits is a multifaceted relationship that can amplify or modify the individual effects of each factor^[20]^. Given that non-additive effects of N and water addition in terrestrial ecosystems, it is crucial to concurrently evaluate the effects of both factors on leaf functional traits^[21]^. However, the interaction between N and water addition has not, to our knowledge, been assessed in temperate forests^[22]^. Systematic study of the adaptation strategies of leaf functional traits and functional diversity to N and precipitation can enrich our understanding of plant responses and community succession mechanisms^[23]^. Notably, prior research has predominantly focused on understory shrub species, with scant attention to the response of canopy tree species' leaf functional traits to N deposition. Therefore, it is imperative to study forest communities holistically.

Previous studies have primarily relied on understory N and water addition methods, disregarding processes such as N or water absorption and interception by forest canopy layers, which cannot accurately simulate the natural process of atmospheric N deposition and precipitation^[24,25]^. Research has demonstrated that more than 40% of N can be absorbed and intercepted by forest canopy layers^[26]^. Furthermore, traditional studies are limited by the use of traditional understory N and water addition methods, resulting in a lack of research on the effects of N deposition and precipitation on leaf functional traits of tree species in the canopy layer, as well as a comparative analysis between tree species and shrub species. This gap impedes subsequent research on the impact of N deposition and precipitation on the entire forest community. Studying the differences in the effects of canopy N and water addition on leaf functional traits and functional diversity indices of tree species and shrub species is significant in revealing the changing trends and response mechanisms of forest ecosystem structure and function under the background of climate change. By introducing N, water, or both to the canopy, we investigated: 1) The impact of N addition on the dynamics of leaf functional traits, and 2) whether N and water additions have combined effects on these parameters.

## Materials and methods

The research site is in Jigongshan (JGS) National Nature Reserve (31°46'−31°52' N, 114°01'−114°06' E), Xinyang City, Henan Province, China. There are four distinct seasons here, with light, heat, and water in the same period. Spring temperatures fluctuate greatly, summer is hot and rainy, autumn is cool with small temperature differences, and winter is long and cold with sparse rain and snow. If divided into four seasons based on the average temperature of the climate, the winter and summer seasons are 125 and 115 d respectively, and the spring and autumn seasons are 65 and 60 d respectively. The annual average solar radiation in JGS is 4,928.70 MJ·cm^−2^, with a total sunshine duration of 2,063.3 h and a sunshine percentage of 47%. The annual average temperature is 15.2 °C, the extreme maximum temperature is 40.9 °C, the extreme minimum temperature is −20.0 °C, and the daily average temperature is stable at or above 10 °C, with an active accumulated temperature of 4,881.0 °C. The frost free period is 220 d. The average annual precipitation is 1,118.7 mm.

### Experimental design

**Figure 1:**
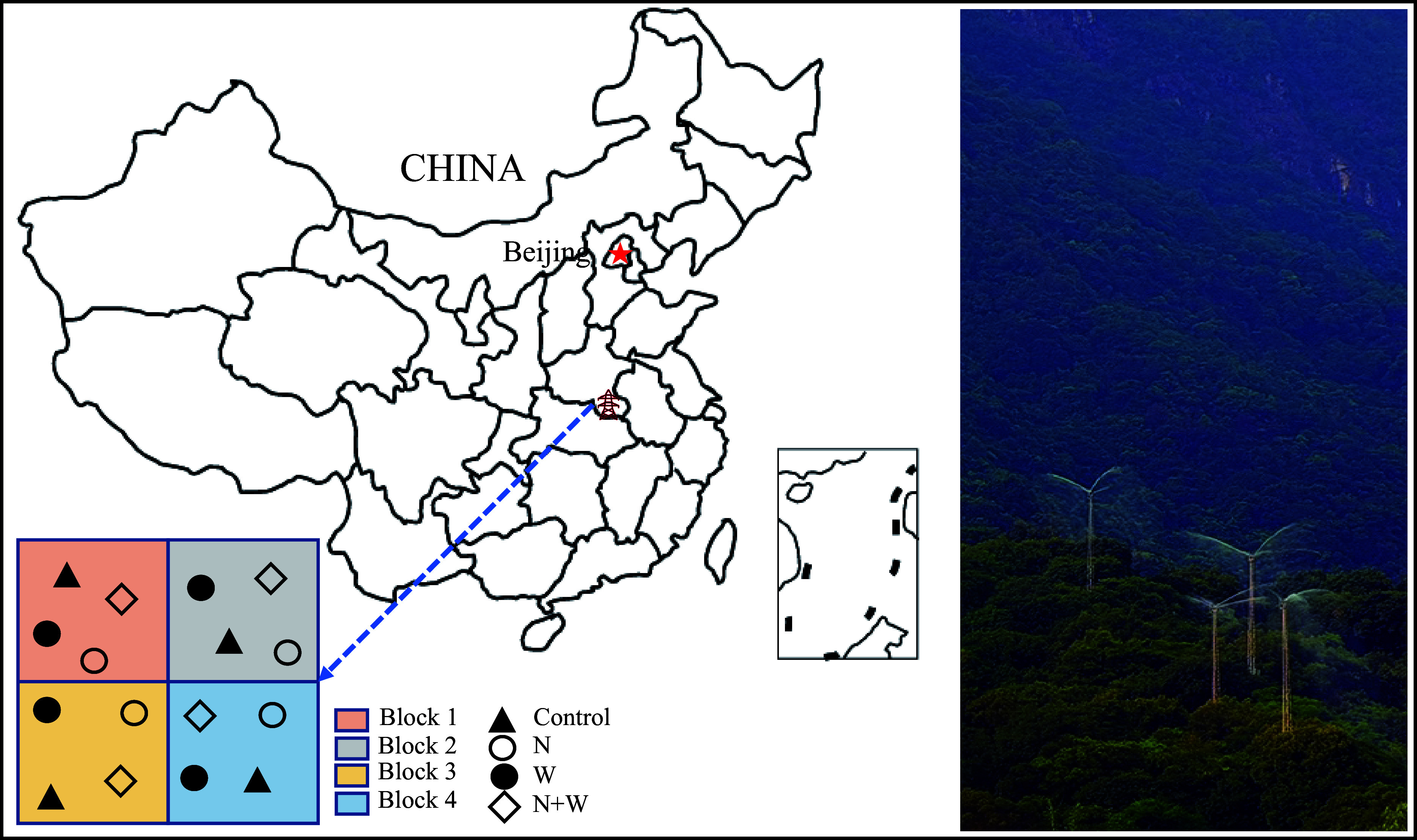
Location of experimental sites and layout of treatment plots at each site and photos of canopy nitrogen and water increase.

We have established a complete dual factor random block design that includes four blocks. The vegetation, terrain, soil, and other environmental factors within the four blocks are relatively consistent. Each block has been randomly assigned with four treatments (907 m^2^ per plot), including control (CK), canopy N addition (addition N 25 kg·ha^−1^·yr^−1^), canopy water addition (addition of water equivalent to 30% of the local precipitation, 336 mm·yr^−1^), and canopy N and water addition. A 20-m buffer zone is established between any two treatments, and a cement board with a depth of 50 cm is buried between the buffer zones to prevent mutual contamination between the treatments (Fig. 1).

In 2012, an iron tower with a height of 35-m (5−8 m above the canopy) was erected in the middle of each treatment except for CK, and the base of the tower was reinforced with cement to enhance stability. A 360-degree rotating nozzle is installed at the top of the tower, with a spraying range of 17 m, ensuring that N solution and water are evenly sprayed within the treatment area. The treatment process began in 2013 and started in April each year, with final treatment in October^[27]^. The required solution for treatment is sourced from the collected local rainwater. In case of insufficient rainwater, water is taken from local lakes. Before canopy addition of N, it is necessary to test the solution (collected rainwater or local lake water) and then add high-purity NH_4_NO_3_ to achieve the target N concentration. The canopy N addition treatment should be carried out on a sunny, windless evening or early morning in the middle of the month (7 times a year) to avoid uncertainties caused by evaporation and local rainstorm. Unlike the canopy addition of N, to prevent surface runoff caused by heavy rainfall, canopy water addition is distributed once a week (four times a month, a total of 28 times from April to October). Canopy addition of N and water is a combination of the two.

### Collection of leaf and soil samples

The field sampling and collection of leaf functional trait data within the sample plot were conducted in early August 2022. Based on vegetation surveys, six dominant species were identified as the research objectives (Table 1). For each tree species, 15−20 adult trees with relatively consistent tree height, trunk that growth fine favorable, and no severe deformation or disease were selected. For each individual, 15−30 mature, healthy, and complete leaves were collected from the upper, middle, and lower layers of each individual's canopy. When collecting, use high branch scissors to collect well growing branches from different directions of each individual and at various vertical heights of the canopy. Branches of different ages were identified based on the node rings formed by bud scales on the branches. Harvest fully unfolded leaves from the current year's branches and promptly seal them in an envelope.

**Table 1:** Sampled dominant tree species in canopy and shrub layers in the warm temperate zone.

Life form	Species names	Family	Genera	Abundance
Tree	*Liquidambar formosana* Hance	Hamamelidaceae	Liquidambar	84
	*Quercus acutissima* Carruth	Fagaceae	Chestnut	464
	*Quercus variabilis* Bl	Fagaceae	Chestnut	100
Shrubs	*Celtis sinensis* Pers	Ulmaceae	Celtis	212
	*Lindera glauca* (Sieb. et Zucc.) Bl	Lauraceae	Lindera	212
	*Acer buergerianum* Miq	Aceraceae	Acer	441

When collecting leaves, three samples were randomly selected within the sample plot with a diameter of 5 cm for initial use and soil collection at a depth of 10 cm. Afterwards, all soil samples were mixed in the sample plot evenly and returned to the laboratory for further processing.

### Measurement indicators and methods

The petioles of each sampled leaf were removed using scissors. Then, the fresh weight of the leaves was taken using an electronic balance (with an accuracy of 0.0001 g). A handheld leaf area meter was then used to measure the leaf area. Next, the leaves were dried at 80 °C for 48 h to obtain biomass data. Finally, the dried leaves were crushed with a mortar and other parameters measured. The potassium dichromate external heating method was used to determine the carbon content of leaves, the Kjeldahl N determination method was used to determine the N content of leaves, and the molybdenum antimony resistance colorimetric method was used to determine the phosphorus content of leaves. The calculation formula for indicators is as follows:

1 $ LDMC=\dfrac{Dry\;weight\;of\;leaf}{Fresh\;weight\;of\;leaf} $

2 $ SLA=\dfrac{Leaf\;area}{Dry\;weight\;of\;leaf} $

3 $ EWT=\dfrac{Fresh\;weight\;of\;leaf-Dry\;weight\;of\;leaf}{Leaf\;area} $

4 $ LNC=\dfrac{Leaf\;total\;nitrogen}{Dry\;weight\;of\;leaf} $

5 $ LPC=\dfrac{Leaf\;total\;phosphorus}{Dry\;weight\;of\;leaf} $

The determination of soil NH_4_-N and NO_3_-N content was carried out by extracting 10 g of soil with 100 mL 1 mol·L^−1^ KCL, and the concentration of the extraction solution was determined using a fully automatic flow injection instrument (Bran Luebbe, Germany).

### Calculation of community weighted mean

The species-by-trait (S) matrix was composed of the functional indicators of leaf of dominant species as well as the distribution, number, and abundance of dominant species in the sample plot. CWM (Community Weighted Mean) can be calculated using the following formula:

6 $ CWM=\sum _{i=1}^{S}{P}_{i}\times {X}_{i} $

where *P*_*i*_ represents the relative abundance of plant species *i* in one particular quadrat, and *X*_*i*_represents one functional trait of plant species *i* in one particular quadrat.

### Statistical analyses and graphic production

CWM was calculated using R (version 4.2.3; The R Foundation for Statistical Computing; www.r-project.org). The R package 'FD' used in the analysis process. To examine the effects of different treatments on the functional indicators of leaves in dominant species and on the physical and chemical properties of soil, a one-way analysis of variance was conducted using SPSS (version 22.0; SPSS Inc., Chicago, IL, USA). Two-factor analysis of variance was used to test whether N and water have an interactive effect on functional indicators of leaf of dominant species. The Shapiro–Wilk Test was used to test the distribution of the data, as well as the Welch test for homogeneity of variance. For non-normal distributions or non-homogeneous variances, logarithmic transformation was used until statistical requirements are met. Paired comparison tests (*p* < 0.05) were performed on the mean values of each sample using the Tukey Kramer Test (HSD). Structural equation modeling (SEM) was performed to elucidate which N and water influence leaf nitrogen content (LNC) and leaf phosphorus content (LPC) in dominant species (SPSS Inc. IL, USA). Figures were produced using Origin 2021 software (OriginLab, Northampton, Massachusetts, USA).

## Results

### Effects of N and water addition on leaf functional traits

The interaction between N and water only had a significant impact on the LDMC of *Celtis sinensis* (*p* = 0.017). Canopy addition of water significantly affected the SLA of *Lindera glauca* (*p* = 0.035; Table 2).

**Table 2:** The effects of nitrogen (N), water (W) and the interaction (N + W) of the two on the functional traits of the leaves.

	N			W			N + W	
	F	P		F	P		F	P
LDMC								
*Quercus acutissima*	0.392	0.542		1.043	0.326		1.234	0.287
*Quercus variabilis*	0.32	0.582		1.824	0.202		0.145	0.71
*Liquidambar formosana*	2.855	0.098		2.493	0.121		0.865	0.357
*Celtis sinensis*	0.31	0.581		2.371	0.131		6.187	**0.017**
*Lindera glauca*	2.133	0.151		0.021	0.886		0.548	0.463
*Acer buergerianum*	1.484	0.23		0.614	0.438		0.52	0.475
SLA								
*Quercus acutissima*	0.258	0.62		0.018	0.895		0.337	0.571
*Quercus variabilis*	0.086	0.774		0.022	0.884		3.638	0.081
*Liquidambar formosana*	3.575	0.065		0.016	0.901		1.134	0.293
*Celtis sinensis*	0.904	0.347		0.266	0.609		0.041	0.841
*Lindera glauca*	0.003	0.96		4.715	**0.035**		0.064	0.801
*Acer buergerianum*	0.485	0.49		0.025	0.875		0.882	0.353
EWT								
*Quercus acutissima*	0.046	0.833		1.387	0.26		0.919	0.355
*Quercus variabilis*	0.116	0.74		2.291	0.156		2.48	0.141
*Liquidambar formosana*	0	0.997		2.583	0.115		0.228	0.635
*Celtis sinensis*	0.177	0.676		0.866	0.358		3.415	0.072
*Lindera glauca*	2.788	0.102		2.995	0.091		0.398	0.531
*Acer buergerianum*	1.36	0.25		1.541	0.221		0.029	0.865
LDMC, leaf dry matter content; SLA, specific leaf area; EWT, equivalent water thickness; bold characters indicate significant differences.								

**Figure 2:**
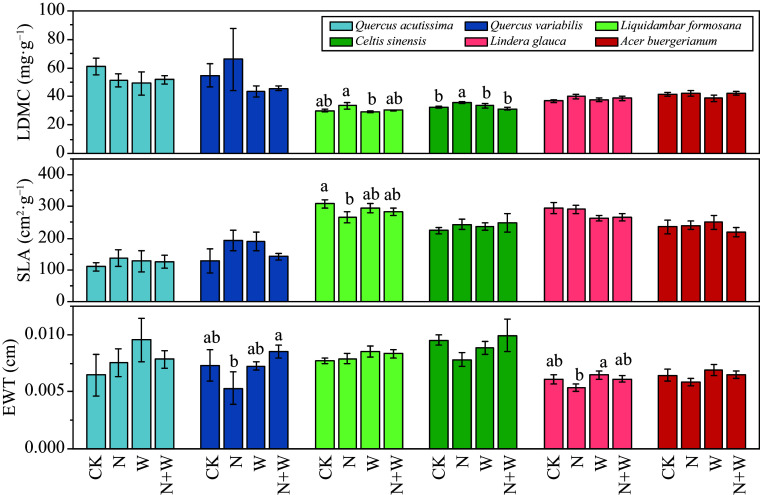
Effect of nitrogen (N) and water addition on leaf functional traits. Treatments included a control (CK), canopy addition N at 25 kg·ha^−1^·yr^−1^ (N), canopy water addition at 30% of the local precipitation (W), canopy N addition at 25 kg·ha^−1^·yr^−1^ and water addition at 30% of the local precipitation (N + W). Different lowercase letters above the error bar (standard error) indicate differences of statistical significance.

The canopy addition of N significantly increased LDMC of *Celtis sinensis*, but significantly reduced the SLA of *Liquidambar formosana* (*p* < 0.05, Fig. 2).

### Effects of N and water addition on leaf chemical properties

Through CWM of the chemical indicators of the leaves, the interaction between N and water significantly affected LNC (*p* = 0.008). In addition, N significantly affected the N/P of the leaves (*p* = 0.041), W significantly affected the LNC (*p* = 0.001) and N/P (*p* = 0.004) of the leaves (Table 3).

**Table 3:** The effects of nitrogen (N), water (W) and the interaction (N + W) of the two on leaf chemical properties.

	N			W			N + W	
	F	P		F	P		F	P
LNC	1.854	0.198		19.842	**0.001**		10.116	**0.008**
LPC	0.027	0.872		0.475	0.504		0.435	0.522
C/N	0.856	0.373		3.03	0.107		2.779	0.121
N/P	5.218	**0.041**		12.613	**0.004**		2.401	0.147
LNC, leaf nitrogen content; LPC, Leaf phosphorus content; bold characters indicate significant differences.								

**Figure 3:**
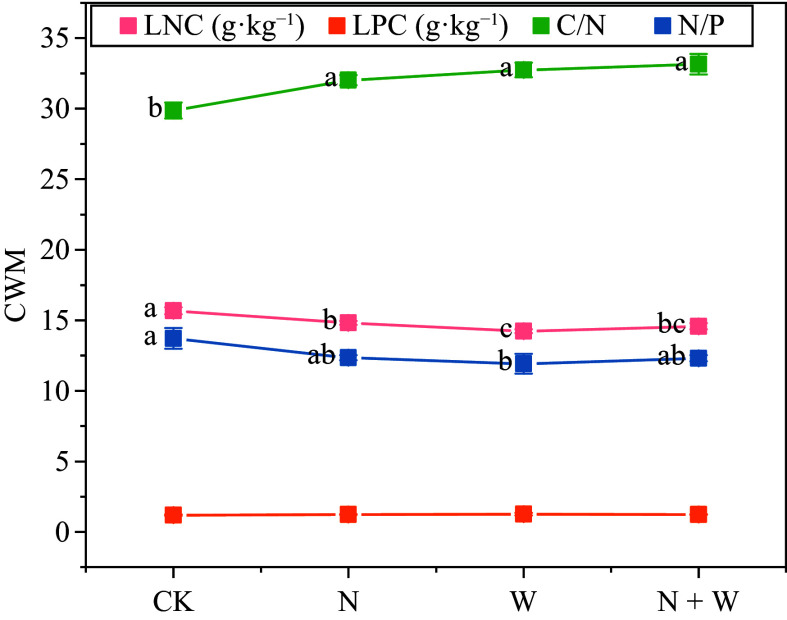
Effect of nitrogen (N) and water addition on leaf chemical properties. Treatments included a control (CK), canopy addition N at 25 kg·ha^−1^·yr^−1^ (N), canopy water addition at 30% of the local precipitation (W), canopy N addition at 25 kg·ha^−1^·yr^−1^ and water addition at 30% of the local precipitation (N + W). Different lowercase letters above the error bar (standard error) indicate differences of statistical significance.

Canopy addition of N, water, and N + W significantly reduced the LNC of the leaves (*p* < 0.05), but significantly increased the C/N of the leaves (*p* < 0.05). Canopy addition of water significantly reduced leaf N/P (*p* < 0.05; Fig. 3).

### Effects of N and water addition on physical and chemical properties in the soil

Canopy addition of water significantly increases the soil moisture content (*p* < 0.05). Canopy addition of N and water significantly increased soil NH_4_-N and NO_3_-N (*p* < 0.05; Table 4).

**Table 4:** Soil moisture content and available N (NH_4_-N, and NO_3_-N) content.

Treatment	Moisture (%)	NH_4_-N (mg·kg^–1^)	NO_3_-N (mg·kg^–1^)
CK	29.01 ± 1.21 b	4.41 ± 0.21 b	17.97 ± 2.92 b
N	28.23 ± 1.04 b	5.11 ± 0.62 ab	21.37 ± 1.64 ab
W	36.98 ± 3.13 a	4.4 ± 0.08 b	15.73 ± 2.92 b
N+W	33.65 ± 2.09 ab	7.61 ± 0.16 a	30.28 ± 3.71 a
CK, control; N, canopy N addition at 25 kg·ha^−1^·yr^−1^; W, canopy water addition at 30% of the local precipitation; N + W, canopy N addition at 25 kg·ha^−1^·yr^−1^ and water addition at 30% of the local precipitation.			

### Pathway of N and water addition on leaf functional traits and leaf chemical properties

**Figure 4:**
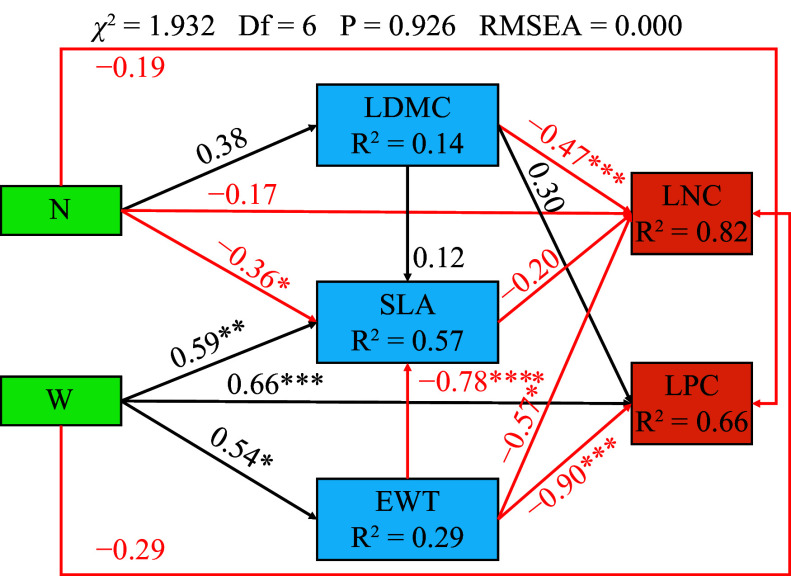
Results of the structural equation model. The arrows represent the hypothesized causal relationships between the variables: N, canopy nitrogen addition at 25 kg·ha^−1^·yr^−1^; W, canopy water addition at 30% of the local precipitation; *, *p* < 0.05; **, *p* < 0.01; ***, *p* < 0.001. Black arrows represent positive effects, and the red arrows represent negative effects. The values next to the arrows are the standardized path coefficients. The values of R^2^ represent the percentage of the response variations explained by the observed variables.

There is a significant direct negative correlation between canopy N addition and SLA (*p* < 0.05). Canopy N addition was positively correlated with LDMC, while LDMC is significantly negatively correlated with LNC (*p* < 0.001) . Canopy water addition is directly and significantly positively correlated with SLA (*p* < 0.01), EWT (*p* < 0.05), and LPC (*p* < 0.001). Moreover, canopy water addition showed an indirectly significant negative correlation with LNC (*p* < 0.001) and LPC (*p* < 0.01) by increasing EWT (Fig. 4).

## Discussion

### Effects of N and water addition on leaf functional traits

In the dynamics of plant growth, factors such as the efficiency of resource acquisition and utilization play crucial roles, while light capture and light energy utilization efficiency are mainly influenced by leaf traits^[28]^. Typically , high SLA coupled with low LDMC indicate that plants is adept at harvesting light and utilizing resources, enabling rapid nutrient acquisition. Conversely, plants with low SLA and high LDMC indicate that plants have lower resource utilization efficiency^[29]^. Our results revealed that the canopy addition of N only significantly decreased SLA of *Liquidambar formosana* (*p* < 0.05, Fig. 2). This indicates that a diminished efficiency in leaf resource utilization when N is added, with a consequent increase in nutrient investment for leaf construction. The likely reason is that when N is abundant, the plants absorb substantial amounts of ${\text{NO}^-_3} $ and ${\text{NH}^-_4} $, which, if not promptly utilized, accumulate in the vacuoles^[30]^. At this time, it is the growing season, mainly consuming nutrients absorbed by plants. Thus, the ${\text{NO}^-_3} $ and ${\text{NH}^-_4} $ stored in vacuoles may serve as ionic and osmotic regulators, modulating the nutrient uptake of *Liquidambar formosana* and leading to reduced efficiency in leaf resource utilization under N-enriched conditions.^[31]^. Additionally, assimilating ${\text{NO}^-_3} $ and ${\text{NH}^-_4} $ requires more energy consumption^[32]^. To conserve energy, *Liquidambar formosana* reduces its leaf area and specific leaf area under nitrogen treatment , thereby reducing C loss. In addition, the experiment revealed that the quantity of *Liquidambar formosana* leaves under N treatment is relatively high , leading to a larger total photosynthetic leaf area. Therefore, a higher LDMC is required to decrease C loss, which in turn results in a relatively small SLA^[28]^. This could be interpreted as a trade-off strategy employed by *Liquidambar formosana* to adapt to the N environment.

Significant differences in leaf functional traits exist among tree species, as well as among different families^[33]^. In natural communities, these differences are crucial for species coexistence and community assembly^[34,35]^. The canopy addition of N and water did not have a significant effect on the morphological characteristics of the leaves of the remaining dominant species. This may be due to inherent differences between species. In natural communities, differences in functional traits of plant leaves are considered an important prerequisite for species coexistence and community construction^[34,35]^. These differences can reflect the various adaptation strategies of plants to different environmental conditions. The differences among different tree species may be attributed to the characteristics of plants themselves. There may also be another explanation. Additionally, the treatments in this study began with the canopy, which may have had a greater impact on the leaves compared to traditional understory addition experiments^[25]^. However, this experiment suggests that the absorption rates of N solution or water attached to the surface are extremely low for most dominant species are extremely low for most dominant species, making it difficult to affect their leaves. In addition, the interception of forest canopies can weaken the soil entry effect, further reducing the treatment effect^[24,36]^. The results also indicate that the canopy addition of N did not significantly increase the available N of the soil (*p* < 0.05; Table 4).

### Effects of N and water addition on leaf stoichiometry

The response of leaf stoichiometric traits to environmental changes is highly species-specific, reflecting the diversity in plant physiological adaptations and survival strategies^[4]^. N and P are critical components that not only inform us about a leaf's photosynthetic potential and resilience to stress but are also sensitive to environmental influences^[37,38]^. Our experimental findings demonstrate that adding N to the canopy significantly lowered the LNC of the dominant species (*p* < 0.05; Fig. 3). Although N addition increased the N uptake of vegetation communities^[39]^, it was mostly used to increase aboveground living biomass, resulting in a significant decrease in LNC. Additionally, our study revealed a notable rise in the leaf C/N ratio (*p* < 0.05; Fig. 3), suggesting alterations in the leaf's elemental composition.

The experiment also found that the canopy addition of water and N-water interaction significantly reduced leaf LNC (*p* < 0.05; Fig. 3). This suggests that the impact of adding water to leaf LNC is consistent with the impact of adding N. This may be due to two separate or combined reasons. In the JGS forest ecosystem, there is a scarcity of both N and water. The addition of water to the canopy can boost nutrient uptake and enhance photosynthesis, leading to increased carbon storage. This process requires substantial N consumption, which could explain the reduced LNC. Previous research has indicated that water addition to the canopy has a more pronounced impact on plant fine roots compared to N addition^[40]^. Water supplementation can lead to less fine root fineness, necessitating a greater N investment to satisfy the nutrient absorption demands of these fine roots, which, in turn, could lead to a decrease in leaf LNC.

The variation in LPC is a multifaceted outcome influenced by soil resources and plant phylogeny^[41]^. Despite minor variations in soil phosphorus availability (Table 3), LPC in leaves is associated with their developmental stage and subject to interannual fluctuations^[42]^. Our study revealed that the experimental treatments exerted no substantial impact on leaf LPC (Fig. 3). This lack of significant change may be attributed to the fact that N and water supplementation to the canopy led to an increase in aboveground biomass, which in turn diluted the LPC on a per-unit-area basis. Furthermore, due to the different charge of ${\text{NO}^-_3} $ and ${\text{NH}^-_4} $, they have different effects on the absorption of other nutrient ions by plants. Typically, ${\text{NH}^-_4} $ tends to enhance the uptake of phosphorus (P) in plants, whereas ${\text{NO}^-_3} $ tends to suppress it. Given that the ratio of these two forms of available nitrogen in the soil was balanced (Table 4), their presence did not markedly affect the LPC.

### Effects of N and water addition on the relationship between leaf functional traits and leaf stoichiometry

Research has shown that canopy addition of N is significantly negatively correlated with SLA (*p* < 0.05), while canopy addition of water is significantly positively correlated with SLA (*p* < 0.01; Fig. 4). This may be because tree species in the canopy typically have a smaller SLA to reduce the impact of low water potential^[43−45]^. Canopy addition of water reduces this impact. Furthermore, a significant positive correlation was found between the canopy addition of water and EWT (*p* < 0.05; Fig. 4). EWT is a measure of plant water status^[11,12]^, which supports the notion that adding canopy water can mitigate the effects of low water potential.

In addition, we found a significant negative correlation between LDMC and LNC. (*p* < 0.001; Fig. 4). Nutrient availability, such as soil available-N, soil moisture, and available-P, may limit warm temperate forest ecosystems^[46]^. N addition can significantly alter plant adaptation strategies, interspecific relationships, and community structure^[39,47]^, increasing vegetation productivity and dominant plant C concentration^[4]^. During the growth period, plants utilize more N to enhance the solidification ability of C. Although N addition increased the N uptake of vegetation communities^[39]^, it was primarily utilized to increase the aboveground biomass, resulting in no significant change or decrease in the LNC content per unit area.

Canopy water addition showed a significant negative correlation with LNC (*p* < 0.001) and LPC (*p* < 0.001) by significantly increasing the EWT of leaves (*p* < 0.05; Fig. 3). Changes in precipitation affect the effectiveness of soil moisture and thermal conditions, regulate soil microbial characteristics, and regulate the supply of soil nutrients. The cycling of materials in ecosystems can be altered through a 'bottom-up' approach^[47]^. Research indicates that when the LNC/LPC of plants are less than 14 (Fig. 3), their growth is easily restricted by N, and vice versa^[41]^. Therefore, water addition treatment will reduce LNC and LPC by increasing the water content of the leaves. Furthermore, several studies have shown a positive correlation between LNC and LPC, which enhance the photosynthetic capacity of plants through synergistic effects^[48]^. The thickening of leaves can decrease the internal water loss in plants and enhance their water retention rate. In response to strong light stress, plant leaves increase the thickness of the stratum corneum and close stomata to prevent further water loss to prevent further water loss. The increase of EWT has to some extent, reduced the photosynthesis of plants, further reducing LNC and LPC.

Natural forests possess a complex community structure, abundant species diversity, and a dense canopy. The study examines the variation of leaf functional traits of dominant tree species in the vertical direction of canopy can further explain the survival strategies and species coexistence mechanism of plants to cope with environmental changes, explore the composition and function of communities, and reflect the indicative role of plants in characterizing the structure and function of ecosystems, it can provide some basis for estimating regional productivity and forest biomass.

## Conclusions

Based on 9 years of continuous processing experiments, it has been demonstrated that the variation in leaf morphological characteristics of dominant plants has significant species specificity. Only *Liquidambar formosana* altered its nutrient acquisition strategy under the treatment of canopy N addition. Under the conditions of N and water addition the community weighted mean analysis showed that, the overall absorption of nutrients and storage of C by plants were enhanced. However, under the background of global climate change, various environmental factors are interrelated and affect each other, which is a dynamic whole. Therefore, the response of plant leaves to climate change is easily affected by the interaction of many factors. Our research only focuses solely on the interaction between N and water. To gain a more comprehensive understanding of the changes in plant leaf functional characteristics under the background of global change.

## Author contributions

The authors confirm contribution to the paper as follows: study conception and design: Li W, Liu M, Li M, Sun R, Zhou T, He Y, Mao J, Liu C, Ma L, Fu S; conceptualization, methodology, software, validation, formal analysis, investigation, data curation, and writing - original draft: Li W; investigation: Liu M, Li M, Sun R, Zhou T, He Y, Mao J, Liu C; conceptualization, investigation, formal analysis, data curation, writing - review and editing, and project administration: Ma L; supervision, resources, and project administration: Fu S. All authors reviewed the results and approved the final version of the manuscript.

## Data availability

The datasets generated during and/or analyzed during the current study are available from the corresponding author on reasonable request.
